# Reverse Conservation Analysis Reveals the Specificity Determining Residues of Cytochrome P450 Family 2 (CYP 2)

**DOI:** 10.4137/ebo.s291

**Published:** 2008-02-09

**Authors:** Tai-Sung Lee

**Affiliations:** Consortium for Bioinformatics and Computational Biology, and Department of Chemistry, University of Minnesota, 207 Pleasant St. SE, Minneapolis, MN 55455

**Keywords:** reverse conservation analysis, P450, CYP 2, SDR

## Abstract

The concept of conservation of amino acids is widely used to identify important alignment positions of orthologs. The assumption is that important amino acid residues will be conserved in the protein family during the evolutionary process. For paralog alignment, on the other hand, the opposite concept can be used to identify residues that are responsible for specificity. Assuming that the function-specific or ligand-specific residue positions will have higher diversity since they are under evolutionary pressure to fit the target specificity, these function-specific or ligand-specific residues positions will have a lower degree of conservation than other positions in a highly conserved paralog alignment. This study assessed the ability of reverse conservation analysis to identify function-specific and ligand-specific residue positions in closely related paralog.

Reverse conservation analysis of paralog alignments successfully identified all six previously reported substrate recognition sites (SRSs) in cytochrome P450 family 2 (CYP 2). Further analysis of each subfamily identified the specificity-determining residues (SDRs) that have been experimentally found. New potential SDRs were also predicted and await confirmation by further experiments or modeling calculations. This concept may be also applied to identify SDRs in other protein families.

## Introduction

Found in all forms of living organisms, the cytochrome P450 superfamily of hemothiolate enzymes are involved in the metabolism of a wide variety of both exogenous and endogenous compounds. In human P450s are key enzymes for metabolizing most drugs and foreign compounds. In fact, over 200,000 chemicals are believed metabolized by P450s. Hence, P450 is of great interest to pharmacologists and toxicologists (Lewis, 1996; Lewis, 2001; Omura et al. 1993; Ortiz de Montellano, 1995).

Identification of the specificity-determining residues (SDRs) of P450s is critical for elucidating the mechanism of substrate/ligand recognition and has immediate and substantial impact on drug design and protein engineering for the P450 proteins. Traditionally, the degree of conservation is used to predict important residues; this concept is widely accepted and has been successfully applied to many systems (Ng and Henikoff, 2003; Sunyaev et al. 2001). However, this approach is not applicable to prediction of SDRs, because the degree of conservation of SDRs is often very low.

The SDRs of each member of the P450 superfamily remain poorly characterized and much effort has been made to accelerate their identification. The concept of substrate recognition sites (SRSs) introduced by Gotoh (Gotoh, 1992) for the P450 2 family (CYP 2) was based on the alignment of mammalian P450s and the structure of a bacterial enzyme, P450cam. The SRS concept has provided an excellent guideline for understanding the basis of P450 specificity and has been used to identify a number of key SDRs in various mammalian P450s have been determined (Wachenfeldt and Johnson, 1995).

Substantial progress has been made in the use of experimental structure information and computation models to better understand specificity (Cruciani et al. 2005; Lewis et al. 1999; Szklarz and Halpert, 1998). Nevertheless, before thorough understanding of 3-D structures of P450s at the atomic level is available, methods are needed to predict the SDRs using sequence alignments or other bioinformatics techniques. Evolutionary analysis—predicting SDRs from the evolutionary history derived from a phylogenetic tree of a given protein family—has been widely used for this purpose (Armon et al. 2001; Landgraf et al. 1999; Lichtarge et al. 1996; Lichtarge and Sowa, 2002; Lichtarge et al. 1997; Madabushi et al. 2002; Sjolander, 1998). Conservation analysis of both paralogs and orthologs has been used to identify the SDRs in bacterial transcription factors (Mirny and Gelfand, 2002). Structural information has been also incorporated into SDR predictions. Yu et al. (Yu et al. 2005) reported a surface patch ranking (SPR) method, which assumes different mutation rates for surface and core residues and uses the evolutionary trace concept to derive the SDRs.

Methods based on conservation analysis or phylogenetic tree information make the basic assumption that the SDRs undergo necessary mutations to compensate for the specificity needed; hence, the residues conserved across the protein family can be distinguished from those conserved only within subfamilies. In such analysis, if a subfamily contains only a single sequence, the conservation of the subfamily is difficult to measure. If the assumption that SDRs have different mutation rates within and outside their subfamilies is true, a fundamentally different view can be employed to identify the SDRs: Since SDRs are under evolutionary pressure to fit the necessary specificity, they may tend to have greater diversity than other positions, especially for paralog sequences. This means that SDRs may have a lower degree of conservation than other positions in a paralog alignment with high similarity. This statement assumes that the degree of evolutionary diversity of non-SDR residues is much less than that of SDR positions, an assumption that is not applicable to distant ortholog sequences.

In this study, the above concept of “reverse conservation analysis” was applied to identify SRSs and SDRs of CYP 2 enzymes. The same concept was then applied to subfamilies of CYP 2, in which both experimentally proven and novel SDRs were identified.

## Theory and Implementation

**Figure 1:**
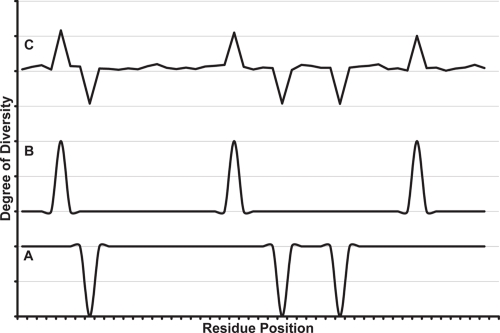
Different degrees of conservation for different cases: **A)** distant orthologs with the same function or specificity; **B)** closely related paralogs with different functions or specificities; **C)** closely related paralogs in a family, with a common function but different specificities (e.g. P450). The down peaks in A correspond to the totally conserved and hence functionally important residue positions. The peaks in B correspond to specificity-determining residues (SDRs). Peaks in C have the same meaning as in A and B. Higher (upward) peaks indicate lower degrees of conservation (higher degrees of diversity). The figure is for illustration of the concept, and arbitrary scales are used.

The concept that the degree of conservation varies in different regions is illustrated in Figure 1. In Case A, which is an alignment consisting of remote orthologs with the same function, a very low degree of conservation is observed for all but functionally important residue positions. In the case of closely related paralog sequences (Case B), a very high degree of conservation is expected for all positions, since the sequence similarity is very high. The SDRs, which must undergo necessary mutations, would have a lower degree of conservation. In Case C, a family of similar proteins with a common function but different specificity is demonstrated. In such cases, e.g. the P450 family, functionally important positions will have a higher degree of conservation whereas SDRs will have a lower degree of conservation. Hence, different strategies should be used for different cases to identify the functionally important residues and SDRs, rather than the currently widely-accepted concept in which only highly conserved residues are thought to be important. When traditional conservation analysis is applied, Case A is usually assumed and much information could be lost.

We assume that the degree of conservation of the CYP 2 family is as in Case C. We used the Rate4Site (Version 2.01) program developed by the Pupko laboratory (Mayrose et al. 2004; Pupko et al. 2002) to calculate the degree of conservation using the empirical Bayesian method. The *S* score calculated from Rate4Site, *S_i_*, which is a measure of the diversity (i.e. the lower the *S* score, the higher the degree of conservation), varies significantly from residue to residue and is difficult to analyze visually. Hence, a window-average of the *S* score, *W_i_*(*N*), is defined as:

(1) $$W_{i}(N)\equiv \left(\sum_{j=i-N/2}^{i+N/2}S_{j}\right)/N$$

where *S_j_* is the Rate4Site score (*S* score) for *j*th position. Currently *N* = *7* is used, which means that for a given *j*th position, the score is calculated by averaging the *S* scores of positions from *j* − *3* to *j* + *3*. The choice of *N* is somewhat arbitrary, although we found that an *N* value of *7* gives curves that are easier to analyze visually. The value 7 could be related to the minimal number of residues needed to form secondary structure units. Using the window-averaged value (*W* score) also assumes that the cooperative mutation around a certain position. Thus the *W* score can be used to identify the SRSs. The *S_i_* score is used together with the *W* score to locate individual SDRs, as will be demonstrated in the results section.

All calculations were performed on a Dell D600 notebook computer with a 1.7GHz Pentium-M CPU and 2 GB RAM. The Rate4Site (Version 2.01) program was downloaded from the www site: http://www.tau.ac.il/~itaymay/cp/rate4site.html.

The CYP 2 sequences were retrieved from Prof. David Nelson’s P450 site at the University of Tennessee (http://drnelson.utmem.edu/CytochromeP450.html). Sixty-nine sequences from 2A, 2B, 2C, and 2D subfamilies were included and the alignment is provided as supplemental information. The numbering system of CYP 2C5 (rabbit) is used throughout the paper.

## Results

### Peak identification

A peak in an arbitrary data set can be defined when its intensity *I* satisfies

(2) I>M+K*SD

where *M* is the mean of the data and *SD* is the standard deviation of the data considered, and *K* is a parameter to control the “strictness” of the peak choice (Mainardi et al. 1997; Todd and Andrews, 1999; Yu et al. 2006). The *S* and *W* scores used here are normalized (Mayrose et al. 2004; Pupko et al. 2002), i.e. the mean is 0 and the standard deviation is 1.0. In the following graphics, we use *K* = 0.5 to demonstrate the peaks.

**Figure 2:**
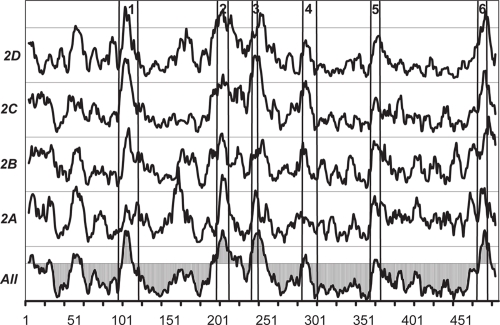
The *W* scores (defined in Eq. 1) for the whole CYP 2 family (marked All) and subfamilies. The score (y) axis is for visual comparison; the units are not shown since they are not relevant (the same applies to all figures). The horizontal axis indicates the residue position. The numbers at the top of the figure and the vertical lines indicate the SRS regions defined by Gotoh (Gotoh, 1992). A cutoff of 0.5 standard deviation unit is shown for the W score of the whole CYP 2 family (marked All). The peaks above this cutoff clearly correspond to the SRS regions.

Figure 2 shows the *W* score, defined in Eq. (1), for the CYP 2 family and its four subfamilies. All six SRS regions defined by Gotoh (Gotoh, 1992) can be identified and are well aligned with the peaks of the *W* score. Although that they are not exactly matched, the consistency between them confirms our assumption on the degree of conservation of SDRs; i.e. Case C in Figure 1. Different patterns (*W* score peaks) were seen for different CYP 2 subfamilies. Each subfamily is discussed in detail below.

### CYP 2A subfamily

**Figure 3:**
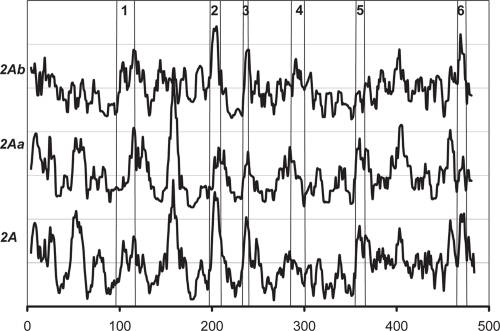
The *W* scores for the CYP 2A subfamily and two subsets of the subfamily.

The CYP 2A subfamily has a *W* score peak located at residue position 150 (Fig. 2). For closer analysis, two clusters from cluster analysis were chosen (based on similarity) as two subgroups; their *W* scores are plotted in Figure 3. These two subgroups have at least 80% identity within the group. The first group, denoted as 2Aa, contains CYP 2A4 (mouse), CYP 2A5 (mouse), CYP 2AA (rabbit), CYP 2AB (rabbit), CYP 2A6 (human), CYP 2A7 (human), and CYP 2AD (human). The second group, 2Ab, contains CYP 2A1 (rat), CYP 2AC (mouse), CYP 2A2 (rat), and CYP 2A9 (golden hamster).

**Figure 4:**
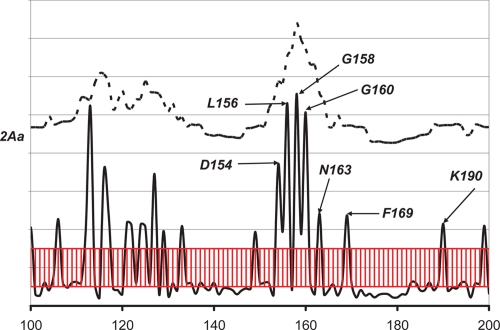
The *S* (solid line) and *W* scores (dashed line) for the 2Aa subgroup of the CYP 2A subfamily. The red region corresponds to the S score region with standard deviation between −0.5 and 0.5. Residues with S scores higher than 0.5 standard deviation unit may be considered as clear peaks.

As seen in Figure 3, the high *W* score peak at the residue position 150 is from subgroup 2Aa. The residues corresponding to this peak may be responsible for the specificity of members in subgroup 2Aa. To investigate the region around this peak, *W* and *S* scores around residue position 150 were calculated (Fig. 4). Few *S* peaks were found, which indicates individual highly diverse positions. These peaks are plotted under the *W* peak, which defines a diverse region around the residue position 150, comprising D154, L156, G158, G160, and N163. The relative structural positions of these residues are very close. A very nearby residue, K190, also has an *S* peak.

The residues corresponding to these *S* peaks are on or near the surface. They may tend to have higher mutation rates and lower degrees of conservation; thus, intuitively, one may conclude that these *S* peaks do not represent SDRs. However, because these peaks are not found in other subfamilies or in the 2Ab subgroup, it is likely that at least some of them are SDRs. Hadidi et al. (Hadidi et al. 1997) reported a single mutation, L156H, will change the reaction selectivity of 2A6 from 7-hydroxylation to 3-hydroxylation of coumarin. Thus, it is evident that the residues found here may cooperatively contribute to the substrate specificity within the CYP 2A subfamily. Further analysis from the structure shows that D154, L156, G158, G160, and N163 are near the end of D helix, whereas K190 is at the beginning of the F helix. This region could be the entrance for the substrate or ligand; hence, these residues may play a “gatekeeper” role to specifically select different substrates or change their entering position and orientation.

### CYP 2B subfamily

**Figure 5:**
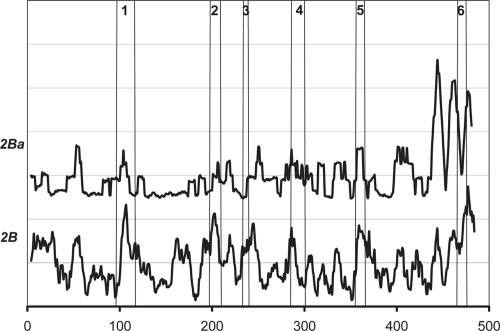
The *W* scores for the CYP 2B subfamily and the 2Ba subgroup of the subfamily.

**Figure 6:**
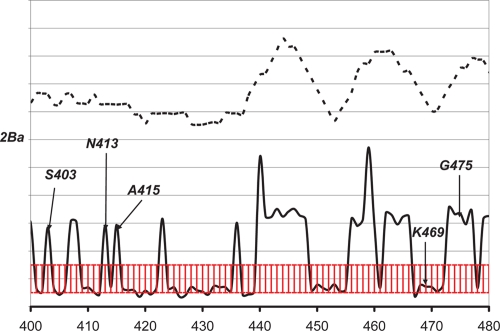
The *S* (solid line) and *W* scores (dashed line) for the 2Ba subgroup of CYP 2B subfamily. The y-axis is not to scale. The red region corresponds to the S score region with standard deviation between −0.5 and 0.5. Residues with S scores higher than 0.5 standard deviation unit may be considered as clear peaks.

The same type of analysis was performed on the CYP 2B subfamily (Fig. 5 and Fig. 6). The 2Ba subgroup has at least 84% identity within the group and contains CYP 2B1 (rat), CYP 2B2 (rat), CYP 2BA (mouse), and CYP 2BK (mouse). Figure 5 clearly shows a case similar to Case B in Figure 1. Figure 6 shows the individual *S* peaks. Von Weymarn et al. (Von Weymarn et al. 2004) reported that G475 in SRS6, along with S403, N413, and A415 (all are non-SRS residues; i.e. not in the SRS regions defined by Gotoh (Gotoh, 1992)) are SDRs, whereas K469 is not an SDR. This is in contrasted to the previous thought for CYP 2B1 and CYP 2B2. These findings clearly agree with the *S* peaks in Figure 6.

The *W* and *S* peaks in the region between residue 456 and 466 correspond to an exposed loop. This variable region may be not related to substrate specificity, although it may be an extension of SRS6. On the other hand, the *W* peak around the residue position 445 may correspond to a specific function, considering that all *S* values are relatively large and the residues are not on or near the surface. Although no experimental evidence has been reported, we are conducting further computational modeling work to study the possible effects of these residues on substrate specificity.

### CYP 2C subfamily

**Figure 7:**
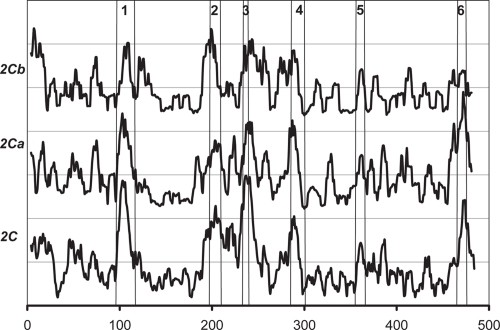
The *W* scores for the CYP 2C subfamily and the 2Ca and 2Cb subgroups of the subfamily.

**Figure 8:**
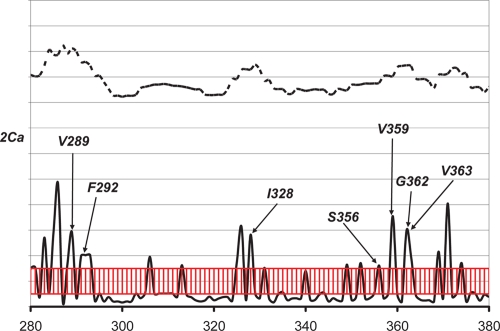
The *S* (solid line) and *W* scores (dashed line) for the 2Ca subgroup of CYP 2C subfamily. The red region corresponds to the S score region with standard deviation between −0.5 and 0.5. Residues with S scores higher than 0.5 standard deviation unit may be considered as clear peaks.

The *W* score of CYP 2C subfamily and the *W* and *S* scores of the 2Ca subgroup (90% identity), containing CYP 2C5 (rabbit), CYP 2C8 (human), CYP 2CK (crab-eating macaque), CYP 2C9 (human), CYP 2CI (human), and CYP 2CI (human), are shown in Figure 7 and Figure 8, respectively. Overall, the pattern of *W* scores of the CYP 2C subfamily is similar to those of other subfamilies, except for the region between residues 100 and 200: in this region, the CYP 2C subfamily shows relatively low *W* score compared to all other subfamilies, which may suggest a different recognition mechanism in this region for CYP 2C.

Niwa et al. found that residues 289, 292, and 328 of CYP 2C9 are essential for the recognition of substrate in CYP 2C9 (Niwa et al. 2002). Kerdpin et al. showed the possible contribution from residues 362, 359, 362 and 363 (Kerdpin et al. 2004). All of these residues clearly correspond to the *S* peaks in Figure 8.

### CYP 2D subfamily

**Figure 9:**
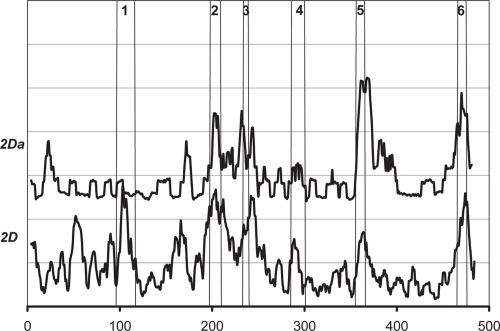
The *W* scores for the CYP 2D subfamily and the 2Da subgroup of the subfamily.

The *W* score for the CYP 2D family is shown in Figure 9. The *W* score of the 2Da subgroup, containing CYP 2D9 (mouse), CYP 2DA (mouse), and CYP 2DB (mouse), shows a manifest example of Case B in Figure 1. So far, we did not find experimental data that can be explained by reverse conservation analysis of the CYP 2D subfamily.

### CYP 2 family

A *W* peak is also observed for all four CYP 2 subfamilies between the residue positions 45 and 62 (Fig. 2). This region is located between the membrane anchor region and the A helix. We are not aware of any experimental evidence of SDRs in this region. Spatially, it is near SRS6 (around residue position 470) and could, together with residues of SRS6, contribute to substrate specificity.

## Discussion

The results from the CYP 2 subfamily indicate that the concept of reverse conservation analysis works well for identification of SRSs and SDRs. All SRSs were identified and several experimentally reported SDRs were well aligned with *S* peaks. However, this method should be used with caution, keeping its assumptions and limitations in mind.

The concept of reverse conservation analysis assumes that the SDRs are under evolutionary pressure and therefore have a lower degree of conservation than other residue positions. Therefore, other factors that could cause a lower degree of conservation should be also considered before a conclusive identification is made. One example is that the surface and core residues normally are considered to have different rates of mutation (Yu et al. 2005). In addition, the correlation of mutation rates between intra-protein residues and residues at the protein-protein interface (Pazos et al. 1997; Sjolander, 1998) should be taken into account, although this is very difficult with alignment-based methods.

As Yu et al. (Yu et al. 2005) pointed out, mutation of SDRs most likely is cooperative among a group of spatially neighboring residues. The analysis here did not consider such cooperative relationships among the SDRs, although a possible case is observed in the CYP 2A subfamily, where D154, L156, G158, G160, N163, and K190 could be a group of residues that cooperatively specify the substrate at the entrance.

Although reverse conservation analysis can detect SDRs by identifying residues with relatively low degrees of conservation, residues with high degrees of conservation cannot be judged as non-SDRs or unimportant. These residues could be functionally important or could play a role in specifying the substrate in one subgroup but not in others. For example, a residue position could be responsible for specifying the substrate for the whole CYP 2 family and thus could be totally conserved in CYP 2, but might have a very low degree of conservation in the P450 superfamily. The different patterns of *S* and *W* curves are noticeable in the results for different CYP 2 subfamilies and subfamilies. The concept of reverse conservation analysis should be carefully applied to different levels of groups (superfamilies, families, subfamilies, or certain groups), just as in Evolutionary Trace methods (Lichtarge et al. 1996).

Reverse conservation analysis should be only applied to alignments with highly similar paralogs. P450 is ideal since it has many similar sequences with different substrate specificities. Even though the CYP 2 sequences used in this paper are from different species, they can be treated as at least “pseudo-paralogs,” since their identity is very high and these sequences are thought to have evolved from the same ancestor (Lewis, 1996; Lewis et al. 1999). The method may be also applied to other systems with such characteristics, such as the kinase superfamily and families. However, as the results of Reverse conservation analysis are highly dependent on the sequences/alignments chosen, one should be careful when choosing alignment and explaining the results. For example, as shown in Figure 5, the result of Reverse conservation analysis on CYP 2B family loses almost all SRS regions as it is understandable in that those SRS regions are conserved within the CYP 2B family.

Most of methods to predict the effects of mutations are based on the degree of conservation (Ng and Henikoff, 2003; Sunyaev et al. 2001; Tchernitchko et al. 2004). They likely will give false-negative results for SDRs, especially when a set of paralog sequences are used. Reverse conservation analysis could be a complementary tool for these mutation prediction methods.

In conclusion, this study proposed conservation analysis in a reverse way and applied this concept to the P450 family 2 (CYP 2). The results of experimentally identified SDRs agree surprisingly well with the peaks of *S* and *W* scores from the reverse conservation analysis, which supports the assumption that the degree of conservation should be lower for SDRs in an alignment containing highly similar paralog sequences. Similar analysis likely can be applied to other systems to identify their SRSs and SDRs.
